# Operations for Rupture of the Perineum and Its Consequences

**Published:** 1869-08

**Authors:** J. F. Miner


ART. II.—*Operations for Rupture of the Perineum and its consequences.* BY J. F. MINER, M. D.

Rupture of the perineum, during the passage of the fœtal head, is not of infrequent occurrence, and is an accident which no ingenuity has yet been able, in all cases, to wholly prevent. The common direction to "support well the perineum" during the last pains of labor, is all very well, but wholly inadequate to prevent laceration. This is applicable to all cases of labor, and, in the majority of instances, is quite sufficient; but, in most cases of great danger to the perineum, it cannot be safely depended upon. Speaking of the best methods of preventing such occurrence, (a digression from the main subject,) it may be said, that nothing is so efficient as lateral incisions, by which means alone can rupture of the perineum be prevented in cases where there is great disproportion between the size of the fœtal head and the vaginal outlet. These lateral incisions may be considerably extended by the passage of the fœtal head, and no great harm come of it. They unite again, and when the union is complete no ill effects follow. Chloroform is often, also, a great protection, by its relaxing properties and relief of spasm of the muscles.

Perineal rupture may vary very greatly in extent. If it involve only a limited portion of the anterior edge, it is of but little consequence, and may be wholly disregarded. If, however, it extend backwards through, or nearly through the entire structure, there will be great risk that, if nothing be done, the patient will hereafter suffer from prolapsus of one or more of the pelvic viscera. If the sphincter of the bowel is ruptured, a still more severe injury, it may result in incontinence of fæces. In all these cases, except those first mentioned, in which the laceration is trivial, it is very desirable that the rent be closed.

The first question, and a very important one, is, When shall this be done? Should opportunity occur, *immediately after delivery.* The edges of the tear should be well cleansed, and secured in apposition by deep sutures. It is quite possible that the bruised, lacerated condition of the parts, the presence of lochial discharge, and the state of the patients general health, may interfere with, retard, or even prevent the union; but, if so, nothing is lost by the attempt having been made. Certain it is, that in very many cases this effort, if well directed, will be successful. If these remarks should impress practitioners in any degree with the importance of this proceeding, our prime object will be gained, for abundant observation leads to the belief that it is almost wholly neglected, and that perineal rupture is left to take care of itself, or its treatment postponed until some months later, when the parts have healed, the lochia is absent, and the necessity of interference more urgent. Many of the slighter ruptures, which may with some degree of propriety be left to themselves, are still much more satisfactorily treated by bringing the edges carefully in contact and retaining them with sutures; the parts are thus restored most perfectly.

Should no operation have been performed immediately after the accident, or should it have failed to secure union, the patient will subsequently come under treatment complaining of prolapsus, or inability to retain the fæces, or both; and to these prime ailments will be added many lesser ills, all springing from or depending upon these. Extensive rupture of perineum often gives rise to the worst forms of prolapse; and the value of the operations which have been proposed for relief of prolapsus uteri, has been more clearly shown in these cases than in almost any other. If the sphincter ani has been torn through, it will not add greatly to the difficulty of the operation, for it is found that union is as easily obtained in such cases as in the lesser lacerations. If the spincter ani has been ruptured, it is very safe to promise restoration by operation, so that control shall be had of the bowel; but we cannot so positively promise complete restoration of the prolapsus. It is quite certain that prolapsus of the uterus, bladder, or rectum, may be present with perfect integrity of the perineum; and it is also just as certain that rupture of the perineum favors prolapse of the pelvic organs. In cases of long standing, too much must not be expected in restoring the prolapse, for it is not infrequently the case that great temporary benefit is later followed by return of the prolapsed, displaced pelvic vicera. This is sufficiently obvious when we consider how other causes than want of support from below operate to cause descent of the viscera into the vulva. The amount of benefit obtained in such cases will depend upon the mode of operation. If the operator denudes only a narrow strip upon either side, making a thin perineum, little more than integument, comparatively little is accomplished—nothing more than that the womb may thus be prevented from complete relapse. If, on the other hand, the operator carry the denudation high up, the lower two inches of the vagina may be greatly narrowed, and the perineum fully restored, thus providing a very effectual barrier to the descent of the pelvic viscera. It must, however, be confessed, that even with this fully accomplished, the uterus may gradually and slowly work its descent, especially if it had, before operation, attained a complete prolapse.

The operation for making a new perineum is made upon the same general principle, whether the sphincter ani is ruptured or not. The edges are to be freely denuded and united by deep sutures; and there is generally thought to be no more difficulty in closing the rupture when extensive than if small. The first step in the operation is an all important one; the complete denudation of the surfaces designed to be united; and want of this is the frequent cause of complete failure. The kind of suture, and the modes of introducing them, will also influence the result. Silver or metalic suture is thought by many as the best, but silk or other thread will answer; and more depends upon the modes of introduction than upon the material of which they are composed. Quilled suture, deeply introduced and carefully adapted—not drawn tightly enough to produce strangulation, and yet sufficiently tense to insure complete contact—is the plan which seems most satisfactory. The necessity of dividing the sphincter ani has been strongly urged by some surgeons. Where the rupture extends high up the recto-vaginal septum, division of the sphincter may be essential; or, where the sphincter itself has been nearly or quite divided, it might be desirable; but, generally, it is quite unnecessary, and very likely in no case absolutely indispensable. There are difficulties and some dangers attending the operation; they are sufficiently obvious in character, and for the most part easily provided for.

Prolapsus uteri is one of the most common results of ruptured perineum; but it is by no means the only ill effect. When it extends through the sphincter ani and destroys control of the bowel, this is by far the most serious evil, and fortunately this is the most certainly cured by operation. The uterus, when wholly prolapsed, is truly in a very inconvenient, unnatural and exposed situation, and is to be replaced, and at least secured within the pelvis. It may rest down below its natural level, supported by the perineum; and, when it becomes accustomed to this new position, suffer comparatively but little. It seems often to rest thus, much more comfortably than when "skewered" up by any form of pessary; indeed, when its natural supports are relaxed, it generally rests easier upon the collateral supports nature has provided than upon any artificial ones, always themselves sources of irritation and discomfort.

If the idea of early treatment, of uniting the ruptured perineum immediately after injury, has been sufficiently insisted upon, my object is accomplished. If laceration is extensive, quilled suture introduced deeply, and adjusted properly, will generally suffice to insure complete union. It prevents a world of trouble, and every practitioner should be alive to its importance. Stitches may be taken in less severe lacerations, which are also of incalculable service in insuring complete restoration of parts. It is remarkable how, without anæsthesia, sutures may be introduced without pain immediately after laceration, the injury and strain upon the parts having, in a great degree, destroyed usual sensibility, so that little, if any, complaint will be made.

